# A Trespass

**Published:** 1911-09

**Authors:** 


### A Trespass

Some of our readers may have seen the play entitled "Too Much Johnson," and may be inclined to paraphrase it with regard to the trespass of the editor upon the original department of this issue. "We," in pursuance with an agreement made by our predecessor, with the Medical Association of Central New York, are obliged to publish "my" article but otherwise expect to confine our material to the proper department.

